# Obituary

**Published:** 1852-01

**Authors:** 


### OBITUARY.

Died, at Philadelphia, Nov. 10, 1851, Dr. J. M. Wallace, in the thirty-seventh year of his age.

Also in New York, on the 9th of Nov., Dr. J. K. Rodgers, one of the most eminent surgeons of our country.

Also in New York, Nov. 12, Granville Sharpe Pattison, M. D., Prof. of Anatomy in the University of the city of N. Y.

Also in New York, Nov. 21, J. R. Manly, M. D., in the seventieth year of his age.

